# Evaluation of Free Light Chains (FLCs) Synthesis in Response to Exposure to SARS-CoV-2

**DOI:** 10.3390/ijms231911589

**Published:** 2022-09-30

**Authors:** Monika Gudowska-Sawczuk, Anna Moniuszko-Malinowska, Sara Pączek, Katarzyna Guziejko, Monika Chorąży, Barbara Mroczko

**Affiliations:** 1 Department of Biochemical Diagnostics, Medical University of Bialystok, Waszyngtona 15A St., 15-269 Bialystok, Poland; 2 Department of Infectious Diseases and Neuroinfections, Medical University of Bialystok, Zurawia 14 St., 15-540 Bialystok, Poland; 3 Department of Biochemical Diagnostics, University Hospital in Bialystok, Waszyngtona 15A St., 15-269 Bialystok, Poland; 4 2nd Department of Lung Diseases and Tuberculosis, Medical University of Bialystok, Zurawia 14 St., 15-40 Bialystok, Poland; 5 Department of Neurology, Medical University of Bialystok, M. Skłodowskiej-Curie 24A St., 15-276 Bialystok, Poland; 6 Department of Neurodegeneration Diagnostics, Medical University of Bialystok, Waszyngtona 15A St., 15-269 Bialystok, Poland

**Keywords:** free light chain, kappa, lambda, SARS-CoV-2, COVID-19, inflammation

## Abstract

The aim of this study is to assess the synthesis of kappa (κ) and lambda (λ) free light chains (FLCs) in the serum of patients with COVID-19. All the 120 serum samples were collected from patients with COVID-19 and from healthy controls (vaccinated and non-vaccinated against SARS-CoV-2). FLCs, IgG total, IgG4, IgG anti-Nucleocapsid (N), anti-spike S1 receptor binding domain (S-RBD) antibodies and IL-6 were measured according to the manufacturers’ instructions. The concentrations of anti-N IgG, IgG total, IgG4 and IL-6 were elevated in the COVID-19 group in comparison to the vaccinated and non-vaccinated controls. The levels of anti-S-RBD IgG and κFLC were increased in COVID-19 and healthy vaccinated patients when compared to non-vaccinated controls. λFLC concentration was higher in the COVID-19 group than in the non-vaccinated group. The κ:λ ratio was lower in both COVID-19 and non-vaccinated groups in comparison to vaccinated controls. κFLC correlated with all tested parameters (anti-S-RBD IgG, anti-N IgG, λFLC, κ:λ ratio, IgG total, IgG4 and IL-6) except CRP, whereas λFLC correlated with all examined parameters except IgG4. Elevated levels of FLCs in COVID-19 and healthy vaccinated against SARS-CoV-2 patients, as well as the correlation between free light chains with specific anti-SARS-CoV-2 antibodies and IL-6, reflect hyperactivation of the immune system after contact with coronavirus. Furthermore, it seems that serum levels of FLCs might be used as predictive markers of COVID-19. Our findings suggest that free light chains are involved in SARS-CoV-2 infection. However, understanding the exact mechanism requires further investigation.

## 1. Introduction

Severe acute respiratory syndrome coronavirus 2 (SARS-CoV-2) is an enveloped, single-stranded RNA virus, which belongs to the group of coronaviruses [1]. Transmission between people occurs mainly by airborne droplets and consequently leads to coronavirus disease—COVID-19 disease [2]. The clinical presentation of COVID-19 may be extremely diverse and there is a wide range of symptoms of coronavirus: from asymptomatic infection to respiratory distress syndrome and even multi-organ failure. However, the symptoms depend on the immune system response [3,4].

Currently, numerous studies are being carried out and new markers of COVID-19 diagnosis and progression are being collected. C-reactive protein (CRP), D-dimer, prothrombin time (PT), procalcitonin (PCT), lactate dehydrogenase (LDH), interleukin-6 (IL-6), ferritin or serum amyloid A (SAA) are mainly mentioned as potential new diagnostic and prognostic biomarkers of SARS-CoV-2 infection. An ideal biomarker should be minimally invasive, repeatable, specific and sensitive. However, importantly, it is very common that the marker can be associated with several different conditions with similar symptoms [5,6]. Therefore, it has been suggested that a perfect indicator of COVID-19 should be directly associated with immune response against SARS-CoV-2. After contact with SARS-CoV-2, the human immune system produces specific antibodies that recognize the virus and help fight the infection. Firstly (about five days from the moment of contact with virus), there is anti-SARS-CoV-2 IgM antibody production in the early stage of the disease [7]. Then, IgG antibodies are produced. IgG can usually be detected no earlier than 7–10 days after the first appearance of symptoms, but they persist for a long time in human blood [8,9]. In addition, IgG has four subclasses: IgG1, IgG2, IgG3 and IgG4, of which it has been suggested that increased level of IgG4 is associated with a worse course of COVID-19 [10]. Importantly, synthesis of anti-SARS-CoV-2 antibodies also occurs after vaccination intended to imitate a natural infection. However, it should be pointed that vaccine antibodies only target the virus’s spike (S) protein. On the other hand, as a result of the disease, antibodies against S protein and/or antibodies to the nucleocapsid (N) protein may occur [11,12].

As is known, immunoglobulins consist of heavy and light chains. Five types of heavy chains (α, μ, γ, δ and ε) and two types of light chains (kappa (κ) or lambda (λ)) can be found in the structure of each immunoglobulin. Light chains are secreted by B lymphocytes during the synthesis of immunoglobulins and, in normal condition, there is an excess of light chains in comparison to heavy chains. Light chains that are not combined with heavy chains are known as free light chains (FLCs) [13,14]. FLCs are involved in several processes of the immune response, e.g., they are necessary for the regulation of the immune system via alteration of neutrophil migration or apoptosis [15]. Moreover, an additional effect of FLC action may be stimulation of a local inflammatory response by the release of some pro-inflammatory mediators, including IL-6.

We hypothesize that the level of FLCs may be associated with the level of IgG, including subclass 4 and specific antibodies against SARS-CoV-2. Thus, taking the above into account, the aim of this study is to evaluate the synthesis of FLCs and to assess their role as predictive markers of SARS-CoV-2 infection.

## 2. Material and Methods

### 2.1. Subjects

A total of 80 patients with COVID-19 were admitted to the Temporary Hospital of the Medical University of Bialystok. Serum samples were collected from all patients with SARS-CoV-2 infection. The tested group was composed of 64 males (age range: 20.0–88.0) and 16 females (age range: 24.0–85.0 years). Out of these 80 COVID-19 patients, 67 were fully vaccinated against SARS-CoV-2.

The diagnosis of COVID-19 was based on either a positive result of a polymerase chain reaction (PCR) test for SARS-CoV-2 or a positive result of a rapid test for the qualitative detection of SARS-CoV-2 antigen from a nasopharyngeal swab on admission. The results of CRP and PCT of COVID-19 patients on admission in are presented in Table 1.

The control group consisted of 40 healthy volunteers (age range: 23.0–75.0 years), which were divided into two subgroups: fully vaccinated against SARS-CoV-2 healthy group (n = 20), and non-vaccinated healthy group (n = 20). Patients from the control group have never been diagnosed with COVID-19.

All vaccinated study participants (COVID-19 and healthy) received all recommended doses of the vaccine according to its type (two doses of Pfizer-BioNTech, Spikevax (Moderna) and AstraZeneca, or one dose of Johnson & Johnson’s Janssen).

Informed consent was obtained from all patients with COVID-19 and from healthy subjects. The study was approved by the Bioethical Committee at the Medical University of Bialystok.

### 2.2. Blood Sampling

Venous blood samples were taken by vein puncture. Then, samples were separated by centrifugation. Serum samples were aliquoted and frozen at −80 °C until the time of analysis.

### 2.3. Analytical Methods

Serum FLC, total IgG and IgG4 were measured using the turbidimetric method on the Optilite analyzer (The Binding Site, Birmingham, UK) and in accordance with the manufacturer instructions. Reference ranges listed in instructions for κFLC, λFLC, κ:λ ratio, total IgG and IgG4 equal 3.30–19.40 mg/L, 5.71–26.30 mg/L, 0.26–1.65, 6.103–16.160 g/L, 39.2–864.0 mg/L, respectively.

IgG antibodies to the nucleocapsid protein (anti-N IgG) and to the receptor binding domain (RBD) of the S1 subunit of the spike protein (anti-S-RBD IgG) of SARS-CoV-2 were measured according to the manufacturer guidelines using chemiluminescent microparticle immunoassay (CMIA) on the Alinity analyzer (Abbott, IL, USA).

Results ≥1.4 for anti-N IgG antibodies and ≥50 AU/mL for anti-S-RBD IgG antibodies were considered as positive.

Quantitative determination of IL-6 was performed using electrochemiluminescence immunoassay (ECLIA) on Cobas e411 and e601 analyzers (Roche Diagnostics GmbH, Mannheim, Germany). The measuring range was 1.50–5000 pg/mL, and for results below the detection limit the value 1.50 pg/mL was used.

### 2.4. Statistical Analysis

Statistical analysis was performed using Statistica 13.3 (StatSoft Polska, Cracow, Poland). The ANOVA rank Kruskal-Wallis test was used to evaluate the differences between the tested groups. The association between the tested variables was measured using Spearman’s rank correlation test. *p* values < 0.05 were considered as statistically significant.

## 3. Results

The results of specific anti-SARS-CoV-2 antibodies, IgG total and IgG4 in COVID-19 patients and healthy volunteers (vaccinated and non-vaccinated) are presented in Table 2. Serum concentrations of anti-S-RBD IgG antibodies, anti-N IgG antibodies, IgG total, IgG4 and IL-6 differ between the tested groups (ANOVA rank Kruskal-Wallis test: *p* < 0.001, *p* < 0.001, *p* < 0.001, *p* = 0.003, *p* = 0.007, respectively). Post-hoc analysis revealed that the serum concentrations of anti-N IgG antibodies, IgG total, IgG4 and IL-6 were significantly higher in COVID-19 patients than in vaccinated (*p* < 0.001, *p* = 0.050, *p* = 0.037, *p* = 0.041, respectively) and non-vaccinated controls (*p* < 0.001, *p* < 0.001, *p* = 0.019, *p* < 0.005, respectively). The concentrations of anti-S-RBD IgG antibodies were significantly lower in non-vaccinated controls in comparison to the COVID-19 (*p* < 0.001) and vaccinated control groups (*p* < 0.001).

The results of free light chains and κ:λ ratio in COVID-19 patients and healthy controls are presented in Table 3.

Serum concentrations of κFLC, λFLC and κ:λ ratio differ between the tested groups (*p* < 0.001 for all comparisons). Post-hoc analysis revealed that the serum concentrations of κFLC were significantly higher in COVID-19 and vaccinated controls in comparison to non-vaccinated controls (*p* < 0.001 for both). The concentration of κFLC did not differ between the COVID-19 and vaccinated control groups (*p* = 0.576). Serum concentrations of λFLC were found to be significantly higher in the COVID-19 group than in non-vaccinated controls (*p* < 0.001). The concentrations of serum λFLC were similar in COVID-19 and non-vaccinated controls in comparison to the vaccinated group (*p* = 0.192, *p* = 0.076, respectively). Values of κ:λ ratio were higher in vaccinated patients in comparison to non-vaccinated and COVID-19 groups (*p* < 0.001 for both). The value of κ:λ ratio did not differ between COVID-19 and non-vaccinated controls (*p* = 1.000).

Correlations between anti-S-RBD IgG, anti-N IgG, κFLC, S λFLC, κ:λ ratio, IgG total IgG4 and CRP are presented in Table 4. Spearman’s rank correlation test demonstrated that in the total study group κFLC correlated with all tested parameters except CRP. λFLC correlated with all examined parameters except IgG4. Anti-S-RBD IgG and total IgG correlated with each other, κFLC, λFLC, and IgG4. In addition, anti-S-RBD IgG was observed to correlate with IL-6. We also revealed the correlation between anti-N IgG and anti-S-RBD IgG, κFLC, λFLC, IgG total, CRP and IL-6. IgG4 correlated with anti-S-RBD IgG, κFLC and IgG total. The highest correlation coefficient was seen between κFLC and λFLC.

## 4. Discussion

COVID-19 is an infectious, viral disease that affects mainly the respiratory tract. In most cases it resembles a mild form of flu-like disease, but in more severe cases, it may cause complications such as pneumonia or respiratory failure. The development of the disease is caused by the SARS-CoV-2 coronavirus [1,2,3]. During the production of viral proteins in the host organism, they are captured by human leukocyte antigens (HLA). Fragments of viral proteins are presented on the surface of infected cells and are then recognized by blood CD8+ T cytotoxic cells, natural killer (NK) or CD4+ T helper cells. The activation of these cells finally results in the production of many pro-inflammatory cytokines which stimulate the cells’ innate response to fight the SARS-CoV-2 effectively. Moreover, synthesis of cytokines stimulates, for example, B lymphocytes to produce antibodies [16,17,18,19]. During the course of maturation of the humoral response, the so-called class switching occurs. This process leads to the replacement of the production of the earliest class of immunoglobulin, IgM, with other classes, including IgG [20]. However, regardless of the class, all immunoglobulin molecules consist of heavy and light chains. In normal conditions, plasma cells produce and release light chains into circulation with a slight excess compared to heavy chains. This excess constitutes a pool of FLCs [13,14,15]. Currently, the clinical significance of FLC determinations in the course of monoclonal gammopathies is well known, as confirmed by numerous literature data [21,22,23]. The diagnostic and clinical significance of the quantitative determination of free immunoglobulin light chains was also assessed in the serum and cerebrospinal fluid (CSF) of patients with multiple sclerosis (MS) [24,25,26]. Interestingly, it has also been observed that viral infections may lead to increased synthesis of FLCs [27,28]. For example, our previous work revealed that increased level of λFLC in CSF may reflect intrathecal production of immunoglobulins or disruption of the blood-brain barrier in patients infected with tick-borne encephalitis virus [29].

Taking the above into account, we have attempted to evaluate the synthesis of FLCs in patients infected by SARS-CoV-2, healthy patients vaccinated against this coronavirus, and healthy non-vaccinated people. According to our best knowledge, this is the first study that assesses the concentration of FLCs and IL-6 in relation to specific SARS-CoV-2 antibodies in COVID-19 as well as healthy people. Until today there has been only one study in which the analysis of FLC synthesis has been performed on a group of patients with COVID-19. However, contrary to our study, the experiment did not include an assessment of anti-N IgG and anti-S-RBD IgG antibodies. Małecka-Giełdowska et al. revealed that there was difference in the rate of FLC synthesis during SARS-CoV-2 infection and, it should be noted, with a significantly increased production of κ chains [30]. Similar results were obtained in the current study. We revealed that patients with COVID-19 had significantly elevated levels of FLCs in comparison to healthy non-vaccinated people. Moreover, κFLC concentration was lower in non-vaccinated vs. healthy vaccinated people. Interestingly, a correlation was observed between FLCs and IgG total, anti-Spike-Receptor Binding Domain IgG, anti-Nucleocapsid IgG and IL-6. On the other hand, IL-6 level correlated with FLCs and specific anti-SARS-CoV-2 antibodies, and did not with total IgG. Therefore, increased concentration of FLCs may indicate acute phase response and elevated synthesis of anti-SARS-CoV-2 antibodies after contact with coronavirus and after vaccination. Moreover, it is worth mentioning that the κ:λ ratio was significantly elevated in the group of healthy and vaccinated patients. Despite the fact that in healthy vaccinated people the κ:λ ratio was still within the reference range (0.26–1.75), the value of the ratio indicates an excess production of κFLCs. Thus, our results seem to be in accordance with the observations of Małecka-Giełdowska et al. [30]. We speculate that after vaccination, hyperactivation of the immune system is observed and therefore the synthesis of light chains, especially κ increases. This can be explained by the fact that rearrangement of the κ light chains gene precedes the rearrangement of λ light chains. Moreover, in contrast to λ genes, the rearrangement of κ genes takes place normally in almost all peripheral B cells [31]. Then again, however, focusing on the fact that the ratio was nevertheless normal, it should be pointed that our study included only COVID-19 patients with a mild course of the disease. As a result of multiple immunoglobulin synthesis, polyclonal FLCs overproduction was also observed. On the other hand, it has been suggested that in severe COVID-19 the presence of “acute” monoclonal gammopathy reflects hyperactivation of the immune system [32]. Moreover, it has been suggested that immunoglobulin G subclass 4 may contribute to the progression of COVID-19. This can be explained by the fact that IgG4 has lower SARS-CoV-2 neutralizing titres compared to other IgG subclasses [10,33]. On the contrary, we observed that patients with a mild course of COVID-19 also had increased levels of IgG4 [32]. Therefore, we carefully suggest that determination of IgG4 concentration may be an important serological biomarker of SARS-CoV-2 infection which can be used for differentiation between COVID-19 and patients without coronavirus infection.

## 5. Conclusions

This research expands the findings of the relationship between FLCs and COVID-19.

Light chains are used by the human immune system to create immunoglobulin-antibodies that neutralize external threat factors, such as viruses. This study confirms that the concentration of free light chains is elevated in mild COVID-19 patients and in healthy patients vaccinated against SARS-CoV-2. Moreover, a positive correlation between FLCs with total IgG, specific anti-SARS-CoV-2 antibodies and IL-6 may directly reflect an acute immune response against coronavirus. Importantly, IL-6 level did not correlate with total IgG, but only with anti-N and anti-S-RBD antibodies, and FLCs. Thus, the obtained results suggest that FLCs may also be independent and very sensitive biomarkers of SARS-CoV-2 infection. The main advantage is that levels of FLCs undeniably indicate anti-SARS-CoV-2 specific antibody synthesis. However, we hypothesize that the rate of FLCs synthesis may also be associated with COVID-19 progression. Therefore, it is unquestionable that further studies on larger groups of COVID-19 patients with different stages of the disease are needed to confirm the role of FLCs in the pathomechanism of SARS-CoV-2 infection.

## Figures and Tables

**Table 1:** The results of inflammatory biomarkers of COVID-19 patients on admission.

Parameter	Mean	SD	Median	Min.–Max.	Reference Range
CRP (mg/L)	18.39	35.08	6.55	1.00–154.90	0.00–10.00
PCT (ng/mL)	0.06	0.03	0.06	0.02–0.18	<0.50

**Table 2:** The results of specific anti-SARS-CoV-2 antibodies, IgG total and IgG4 in tested groups. Variable tested. Mean ± SD (min–max values).

	COVID-19	Vaccinated Controls	Non-Vaccinated Controls
anti-S-RBD IgG antibodies [AU/mL]	11,881.58 ± 12,032.78 ^C,^ * (0.00–48732.00)	18,672.07 ± 17,327.06 ^C,^ * (705.80–65337.60)	0.09 ± 0.06 ^A, B,^ * (0.03–0.23)
anti-N IgG antibodies [Index]	4.99 ± 4.54 ^B, C,^ * (0.02–11.43)	0.76 ± 1.70 ^A,^ * (0.01–6.27)	0.03 ± 0.04 ^A,^ * (0.01–0.13)
IgG total [g/L]	11.38 ± 2.58 ^B, C,^ * (4.57–17.20)	9.96 ± 1.29 ^A,^ * (7.19–12.20)	9.29 ± 1.62 ^A,^ * (6.44–11.84)
IgG4 [mg/L]	741.41 ± 482.68 ^B, C,^ * (117.30–2574.80)	436.31 ± 243.95 ^A,^ * (107.30–926.60)	441.71 ± 321.77 ^A,^ * (39.60–1274.70)
IL-6 [pg/mL]	8.74 ± 14.22 ^B, C,^ * (1.50–94.90)	2.45 ± 1.90 ^A,^ * (1.50–4.74)	1.60 ± 2.05 ^A,^ * (1.50–3.14)

^A^, COVID-19; ^B^, Vaccinated Controls; ^C^, Non-Vaccinated Controls. * the significant differences between tested groups.

**Table 3:** The results of free light chains and κ:λ ratio in COVID-19 patients and healthy controls. Variable tested. Mean ± SD (min–max values).

	COVID-19	Vaccinated Controls	Non-Vaccinated Controls
κFLC [mg/L]	16.76 ± 5.51 ^C,^ * (5.25–42.50)	17.83 ± 3.03 ^C,^ * (12.10–23.70)	10.25 ± 2.13 ^A, B,^ * (6.28–15.04)
λFLC [mg/L]	16.38 ± 6.17 ^C,^ * (6.32–36.50)	13.22 ± 3.87 (9.24–22.00)	10.26 ± 2.76 ^A,^ * (6.84–18.89)
κ:λ ratio	1.10 ± 0.28 ^B,^ * (0.44–1.94)	1.40 ± 0.24 ^A, C,^ * (0.88–1.77)	1.03 ± 0.22 ^B,^ * (0.51–1.41)

^A^, COVID-19; ^B^, Vaccinated Controls; ^C^, Non-Vaccinated Controls. * the significant differences between tested groups.

**Table 4:** Spearman’s correlations between tested variables in the total study group.

Total Study Group	Anti- S-RBD	Anti-N	κFLC	λFLC	κ:λ Ratio	IgG Total	IgG4	IL-6	CRP
anti-S-RBD r *p*		0.373 <0.001 *	0.434 <0.001 *	0.318 <0.001 *	0.064 0.520	0.261 0.008 *	0.247 0.018 *	0.259 0.006 *	0.094 0.326
anti-N r *p*	0.373 <0.001 *		0.228 0.021 *	0.312 <0.001 *	−0.096 0.334	0.370 <0.001 *	0.152 0.153	0.280 0.003 *	0.084 0.380
κFLC r *p*	0.434 <0.001 *	0.228 0.021 *		0.716 <0.001 *	0.300 0.002 *	0.470 <0.001 *	0.258 0.012 *	0.200 0.044 *	0.171 0.084
λFLC r *p*	0.318 <0.001 *	0.312 <0.001 *	0.716 <0.001 *		−0.369 <0.001 *	0.453 <0.001 *	0.174 0.093	0.224 0.019 *	0.204 0.031 *
κ:λ ratio r *p*	0.064 0.520	−0.096 0.334	0.300 0.002 *	−0.369 <0.001 *		−0.001 0.991	0.124 0.232	−0.057 0.566	−0.020 0.841
IgG total R *p*	0.261 0.008 *	0.370 <0.001 *	0.470 <0.001 *	0.453 <0.001 *	−0.001 0.991		0.354 <0.001 *	0.109 0.276	0.062 0.538
IgG4 R *p*	0.247 0.018 *	0.152 0.153	0.258 0.012 *	0.174 0.093	0.124 0.232	0.354 <0.001 *		−0.020 0.853	−0.138 0.186
IL-6 R *p*	0.259 0.006 *	0.280 0.003 *	0.200 0.044 *	0.224 0.019 *	−0.057 0.566	0.109 0.276	−0.020 0.853		0.377 <0.001 *
CRP R *p*	0.094 0.326	0.084 0.380	0.171 0.084	0.204 0.031 *	−0.020 0.841	0.062 0.538	−0.138 0.186	0.377 <0.001 *	
	**Correlation Ratio (r)**								
	0.000–0.100								
	0.101–0.300								
	0.301–0.500								
	0.501–0.700								
	0.701–0.900								

* statistically significant results (*p* < 0.05).

## Data Availability

The data that support the findings will be available on request under the corresponding author’s e-mail: monika.gudowska-sawczuk@umb.edu.pl.
